# Influence of Distance, Environmental Factors, and Native Vegetation on Honeybee (*Apis mellifera*) Foraging in Arid Shrublands and Grasslands

**DOI:** 10.3390/insects15070543

**Published:** 2024-07-18

**Authors:** Alma Delia Baez-Gonzalez, Mario Humberto Royo-Marquez, Carlos Alejandro Perez-Quintana, Adrián Isaac Hernández-Bernal, Alicia Melgoza-Castillo, Mieke Titulaer, Jose Humberto Vega-Mares

**Affiliations:** 1 Campo Experimental Pabellon, Instituto Nacional de Investigaciones Forestales, Agricolas y Pecuarias (INIFAP), Km 32.5 Carr. Aguascalientes-Zacatecas, Pabellon de Arteaga 20660, Aguascalientes, Mexico; 2 Campo Experimental La Campana, Instituto Nacional de Investigaciones Forestales, Agricolas y Pecuarias (INIFAP), Km 33.5 Carr. Chihuahua-Ojinaga, Cd. Aldama 32910, Chihuahua, Mexico; 3 Facultad de Zootecnia y Ecologia, Universidad Autonoma de Chihuahua, Perif. Francisco. R. Almada Km 1, Chihuahua 33820, Chihuahua, Mexico

**Keywords:** *Apis mellifera*, *Koeberlinia spinosa*, *Mimosa aculeaticarpa*, *Neltuma glandulosa*, bee colonies, bees in arid Mexico, bee foraging and environmental parameters, bees and native vegetation

## Abstract

**Simple Summary:**

This study determined the influence of distance, environmental factors, and native vegetation on honeybee (*Apis mellifera*) foraging in arid shrublands and grasslands in Northern Mexico. Apiary distance from inflorescence sites was not a significant factor in foraging, while the honeybee response to environmental factors was influenced by apiary location and landscape. Air and minimum temperature, wind velocity, and relative humidity affected foraging in open areas in shrublands but not in grassland areas surrounded by hills (1820 to 2020 amsl). Nights with a minimum temperature of <20 °C increased foraging activity during the day. Minimum temperature is a driving variable that can be considered for further studies on the impact of climate change on bee activity. High intensity of honeybee foraging was observed in allthorn (*Koeberlinia spinosa*) and wait-a-minute bush (*Mimosa aculeaticarpa*) in shrublands and honey mesquite (*Neltuma glandulosa*) and wait-a-minute bush (*Mimosa aculeaticarpa*) in grasslands. The findings and baseline data contributed by this study may be used to identify suitable environments for increasing apiary productivity and other agricultural and ecological benefits.

**Abstract:**

This study determined the influence of foraging distance, environmental factors, and native vegetation on honeybee (*Apis mellifera*) foraging in arid shrublands and grasslands in Northern Mexico. Apiary distance from inflorescence sites did not have a significant influence on the intensity of foraging. Apiary location and landscape were decisive factors in the response of honeybees to environmental factors. Air temperature, minimum temperature, wind velocity, and relative humidity explained foraging by 87, 80, 68, and 41% (R^2^), respectively, in shrubland sites in open landscapes but had no significant influence on foraging in the grassland sites in a valley surrounded by hills (1820–2020 amsl). Nights with a minimum temperature of <20 °C increased foraging activity during the day. Minimum temperature, which has the least correlative influence among climate elements, can be used to determine climate change’s impact on bees. The quantity of available inflorescence explained the foraging intensity by 78% in shrublands and 84% in grasslands. Moreover, when honeybees depended mainly on native vegetation in grasslands, the quantity of inflorescence explained the intensity of foraging by 95%. High intensity of honeybee foraging was observed in allthorn (*Koeberlinia spinosa*) and wait-a-minute bush (*Mimosa aculeaticarpa*) in shrublands and honey mesquite (*Neltuma glandulosa*) and wait-a-minute bush (*Mimosa aculeaticarpa*) in grasslands. The findings and baseline data contributed by this study may be used to identify suitable environments for increasing apiary productivity and other agricultural and ecological benefits.

## 1. Introduction

Bees and other insects provide pollination services that sustain terrestrial food webs [1]. Around 75% of human nutrition requires the work of pollinators [2]. Pollination is also crucial to plant biodiversity, as 60 to 90% of plant species in the world require it to reproduce [3]. With the increasing global demand for crops dependent on pollination, the essential services provided by bees for agricultural productivity and ecosystem health have grown in importance [1,4].

*Apis mellifera* L., the European honeybee, is the bee species with the largest geographic distribution in the world and is also the most valued due to the current high demand for honey [3,5]. As generalist pollinators of wild plants and agricultural crops, honeybees play a crucial role in maintaining plant diversity and food security, contributing one-third to food production [3,6,7]. A US study estimated the service honeybees provide in pollinating agricultural crops at over USD 14.6 billion [2]. In Mexico, the value of honeybee pollination of cultivated plants is 20 times greater than the value of honey production [8]. Aside from being the main pollinators of flowering plants [7], honeybees serve as the primary and secondary pollinators of many other plants, including highbush blueberry (*Vaccinium* sect. *Cyanococcus*), apple (*Malus pumila*), pear (*Pyrus*), almond (*Prunus dulcis*), and cantaloupe (*Cucumis melo* var. *cantalupensis*) [9]. Mexican studies have shown that the pollinating role of honeybees is variable [5]; for instance, they are important pollinators for avocados but not the most effective for tomatoes, habanero peppers, and coffee. As globally managed pollinators having diverse interactions with the environment, they serve as sampling devices of their surroundings and hence can be used to monitor climate change and other emerging threats [1,10].

The high demand in the international market for high-quality honey produced in Mexico [8] has made the country one of the leading producers and exporters of honey, with an annual production of 62,320 tons of honey, generating a foreign exchange of USD 93,725 million [11]. Mexico ranks sixth in honey production and third in exports globally [3,11,12]. The diversity of climates in Mexico (tropical, subtropical, and temperate) has allowed the growth of the beekeeping industry throughout the country; apiculture has become an activity of great economic, social, and ecological importance and a major source of employment and income [3,5,13]. Most of the approximately 43,000 beekeepers [5,8] are small-scale producers, for whom the sale of honey and wax represents an important part of their income. Approximately 60% of the bee colonies in Mexico belong to these small-scale beekeepers, who manage 40 hives on average and have a low degree of technification (i.e., use of technology); the remaining colonies (40%) belong to production units of various sizes and degrees of technification [8].

Unfortunately, honeybees are among the managed pollinator species whose populations have suffered significant declines worldwide [1,14] due to biotic and abiotic factors as well as anthropogenic ones [2,3,15,16]. Habitat loss is considered the major contributor to declining bee populations across the world, with urban and agricultural development and the increasing practice of growing monocrops without leaving habitat for honeybees as important factors [2]. The growing lack of diverse nectar and pollen resources within intensively farmed agricultural landscapes [14] and the increasing use of toxic chemical substances in modern agriculture to control weeds and pests and ensure high yields are also detrimental to honeybees [17,18].

In Mexico, a country-level assessment based on 32 years of data (1980–2012) showed a significant decline in the number of beehives, though an increase in average annual yield per hive [6]. The central states of Queretaro, Mexico, and Guanajuato and the northern states of Baja California, Chihuahua, Coahuila, Sonora, Durango, San Luis Potosi, Tamaulipas, and Nuevo Leon particularly showed significantly high rates of reductions in the number of hives (−0.21% to −0.52%). Land-use changes, unfavorable climatic conditions, and political and socioeconomic factors were cited as partially responsible for the reductions, with agricultural expansion seen as the most important driver of ecosystem change in present and future scenarios [6]. On the other hand, a later study based on 2009–2018 data [5] showed a significant increase in the number of hives in 16 states, a significant decrease in only 9 states, and no change in 7 states. The non-homogeneous spatial distribution of hives and the beekeepers’ interest in seeking certain flowering plants to increase the value of honey have resulted in regions with a higher density of bees and others that are underutilized [5].

It is generally known that bee behavior is affected by both in-colony and out-colony factors [9,19]. However, studies on the ecology of *A. mellifera* in Mexico, such as the flora they visit and the quality of their foraging territories, are scarce [5], especially in arid regions. Such information can help determine which areas are more favorable to locate or move apiaries and can contribute to planning management and conservation strategies. Hence, this study has the following objectives: (1) to characterize apiary areas in understudied arid shrublands and grasslands in Northern Mexico; (2) to analyze how honeybees respond to foraging distance, environmental factors, and native vegetation in these areas; and (3) to provide baseline data that may be extended in time and space for honeybee management in arid areas.

## 2. Materials and Methods

### 2.1. General Geospatial Analysis of the Apiary Sites

**Figure 1:**
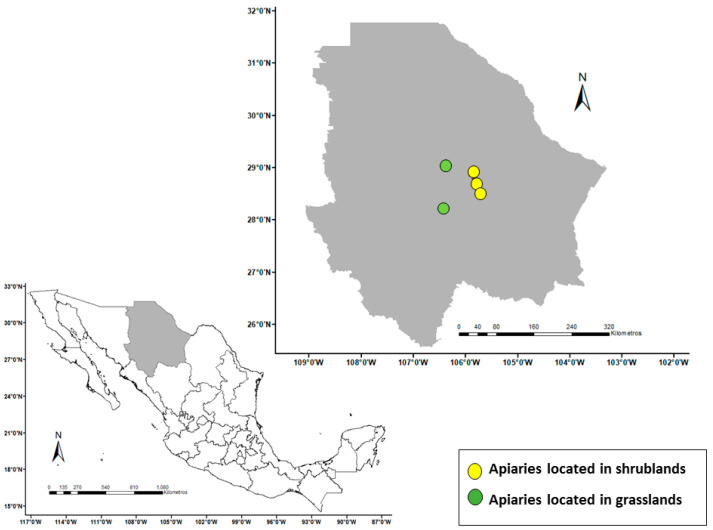
Geographic location of five apiaries in Chihuahua, Mexico.

Five sites with apiaries were observed (Figure 1). Apiaries 1, 2, and 3 were in shrublands and had eight, six, and ten colonies, respectively. Apiaries 4 and 5 were in grasslands and had fifteen and six colonies, respectively. All five sites were in Chihuahua, one of the northern Mexican states with significantly high rates of hive decline [6]. Farmers had established the apiaries close to flowering crops and trees that are known to be foraging sources; however, the study was limited to foraging in relation to native vegetation, as this aspect is understudied [5]. Additional characteristics of the study areas are presented subsequently (Section 3.1 and Section 3.2) as results of the geospatial and botanical characterization phase of the study.

A geospatial analysis, i.e., the use of geographic information that describes an entity in relation to its location [20,21,22,23], was performed on the study sites with the use of several digital platforms. This analysis aims to characterize the location of the apiaries under study, considering some environmental elements, such as topographic conditions, wind velocity, and wind energy, which can have an influence on bee foraging. Part of the analysis was conducted in a geographic information system (GIS) with the ArcGis 10.3 software from Esri, Inc., Redlands, CA, USA [24]. In the GIS, the topographic chart was projected at a scale of 1:50,000, Series I. 1968–1988 [25]. The Google Earth Pro digital platform was also used, with a compilation date of May 2022 and satellite images of the study area taken in September 2021. The Global Wind Atlas 3.0 [26] was used for the analysis of the average annual wind velocity (m s^−1^) and wind energy (watts m^−2^).

### 2.2. Vegetation Characterization

The botanical composition and the floral production in the shrublands and grasslands were characterized every seven days during the spring and early summer period of March–July 2022.

The botanical composition was determined at distances of every 500 m in a ratio of 1500 m from the apiary in two opposite directions [27]. The methodologies of Herrick et al. and Melgoza and Fierro were used [28,29]. In a line transect of 50 points in 100 m, the plant species that intercepted the line in the different strata (herbaceous, shrubby, and arboreal) were counted, along with the bare soil, rock, and organic matter. The intercepted plant species were at or above ground level.

#### Floral Production

In shrublands, the quantification of the floral production was performed using five plants for each species of shrub present along the line transect. The sampling site area was 100 m^2^. Three reference units were selected for each shrub species present in the sampling area. The process consisted of selecting branches in terms of size and morphology as well as foliage and flowering density of the bush to estimate the number of reference units in the bush [30]. In each reference unit, the number of solitary flowers or inflorescences and the number of flowers per inflorescence were counted. The inflorescence counts of three branch samples were added, and the average was obtained. This average was multiplied by the number of reference units detected in each bush. The data on the average number of flowers per bush were used to obtain the quantity of inflorescence that is discussed in relation to forage activity in the subsequent sections.

In grasslands, the plants included grasses and other monocot and dicot herbaceous plants. For any type of plant, 3 to 5 individuals were chosen. Depending on the size of the inflorescence and flowers of species present in the sampling site of 25 m^2^, the count was carried out in situ, in the case of conspicuous flowers, or in the laboratory, where samples were counted with the help of a stereoscopic microscope.

### 2.3. Foraging and Vegetation

The methodology based on Guallpa-Calva et al. [31] was used to measure foraging on the flowering species present in the study area. The samplings were carried out at distances of 250 m, 750–850 m, and 1300–1500 m from the apiaries, as in previous studies [32,33,34]. At least five visual observations of 25 s for each species were made in an area of 1 m^2^ that had flowers. The area in m^2^ refers to the area of the most abundant species in the site that had flowers or those species that were not among the most abundant ones in the area but were frequently visited by bees. A minimum period of 5 s spent by the bee on a flower was registered as foraging. The process was repeated until observations had been made of all species that were flowering at that time. The data were recorded during at least two different days every seven days for each observed species and were analyzed as an average. Plants were assigned values of 1 = less than 0.1 bees m^−2^, 2 = 0.1 to 1 bees m^−2^, 3 = 1 to 2 bees m^−2^, 4 = 2 to 3 bees m^−2^, and 5 = more than 3 bees m^−2^.

### 2.4. Foraging and Environmental Parameters

The following data were collected to determine the influence of environmental parameters on foraging: air temperature (°C), minimum temperature (°C), atmospheric pressure (mbar), wind velocity (km h^−1^), relative humidity (%), and cloudiness. Other data, such as the time and date of observation, were also recorded. The environmental data were obtained from the Google Weather app platform at the time of the field observations.

### 2.5. Statistical Analysis

The two different habitats (shrublands and grasslands) were analyzed separately. In shrublands, 18 samplings were made in Apiary 1 and Apiary 2, and 19 samplings in Apiary 3. In grasslands, 12 samplings were made in Apiary 4 and 18 samplings in Apiary 5. The data were analyzed using generalized linear models in GraphPad software version 5.01, GraphPad Prisms Inc., ^®^ Boston, MA, USA. The established significance level was *p* ≤ 0.05. The analysis focused first on determining the dependence of foraging on each of the environmental parameters. Then, linear regression was also used for the analysis of the quantity of inflorescence as the independent variable, and the intensity of foraging was used as the dependent variable. The intensity of foraging was measured as the number of bees m^−2^ with a minimum period of 5 s spent by the bee on a flower [31]. The quantity of inflorescence and intensity of foraging data were log_10_-transformed, and the normality of the transformed data was tested using the Shapiro–Wilk test and Q–Q plot. At the same time, it was determined if there was any relationship between the distance of the apiaries and the intensity of foraging recorded during the samplings conducted in the five study sites.

## 3. Results and Discussion

### 3.1. Geospatial Characteristics of the Apiary Sites 

#### 3.1.1. Shrubland Sites

Apiary 1, with eight bee colonies, was situated 10 m from a walnut orchard and 300–600 m from irrigated croplands of alfalfa (*Medicago sativa*) in an open area between two hill ranges with altitudes of 1240–1250 amsl. Based on the wind atlas [26], the area had prevailing winds with a southeasterly direction, with an average velocity of 4 m s^−1^ and energy of 99 watts m^−2^.

Apiary 2, with six bee colonies, was located 8.8 km from Apiary 1, also close (200 to 300 m) to irrigated alfalfa croplands, and in an open area between two hill ranges with altitudes of 1240–1250 amsl. The wind energy was 105 watts m^−2^.

Apiary 3, with 10 bee colonies, was 6.8 km from Apiary 2 and 15.6 km from Apiary 1. It was close (50 m) to an area of native vegetation (wetland) and was between hill ranges with altitudes of 1220–1230 amsl. The wind velocity in this site—despite having relatively low annual averages (4 m s^−1^)—presented the highest wind energy (113 watts m^−2^). One peculiarity of Apiary 3 was that it was situated at a distance of 108 m from a perimeter fence, which seemed to have created a specific microclimate in the area.

The climate type of the three apiaries is classified as very hot temperate with a mean annual temperature of 16 °C and 354 mm of mean annual precipitation [35].

#### 3.1.2. Grassland Sites

Apiaries 4 and 5 were located in grassland areas at an average altitude of 1815 amsl. Apiary 4, with 15 bee colonies, was surrounded by agricultural fields where common beans (*Phaseolus vulgaris* L.) were grown and by ravines and hilly pastureland. On the other hand, Apiary 5, with six bee colonies, was not close to any cropland. Both apiaries were on a valley 700–1200 m from a surrounding hill range with altitudes of 1820–2020 amsl. The average annual wind velocity was 5 to 6 m s^−1^, and its energy was 148 and 343 w m^−2^ in Apiaries 4 and 5, respectively.

The climate type of the valley where both apiaries were located is classified as dry temperate, with hot and dry summers with mean annual temperatures of 13 °C and a mean annual precipitation of 378 mm [35].

### 3.2. Botanical Composition 

#### 3.2.1. Shrublands

The botanical composition in the shrublands was dominated by *Larrea tridentata*, *Neltuma glandulosa,* and *Flourensia cernua*, with varying percentages of presence in the sites (Table 1). In Apiary 1, the dominant species were *Neltuma glandulosa*, 50.1%; *Flourensia cernua*, 16.8%; and *Larrea tridentata*, 15.3%. In Apiary 2, the dominant species were *Neltuma glandulosa*, 45.8%; *Flourensia cernua*, 19%; and *Larrea tridentata*, 17.3%. In Apiary 3, *Neltuma glandulosa* represented 48% of the botanical composition; *Flourensia cernura*, 20.7%; and *Larrea tridentata*, 14.4%. The botanical composition showed a wide diversity, with dominant plants that were constant and other plants, mainly some grasses such as *Bouteloua barbata* and *Sporobolus airoides* and some cactaceas such as *Echinocereus stramineus* and *Opuntia engelmannii*, that were not included due to their low contribution to the botanical composition.

All the shrubland apiaries were located on cattle ranches that were extensively exploited. The types of plant species present and their percentages of contribution to the botanical composition may have varied due to the influence of the intrinsic geospatial features of each site and the impact of the grazing management and type of land use applied by the ranchers (i.e., mesquite firewood, medicinal plants, etc.).

#### 3.2.2. Grasslands

Table 2 shows the list of dominant plants in Apiaries 4 and 5 in the grasslands. The dominant species were grasses and other herbaceous plants, while shrubs were minimal. The dominant species in this area were the grass *Bouteloua gracilis* and the shrub *Mimosa aculeaticarpa*. Other species, such as *Muhlenbergia rigida*, *Muhlenbergia repens*, *Panicum hallii*, *Panicum hirticaule*, *Hopia obtusa*, *Artemisia ludoviciana*, *Juniperus deppeana*, *Solanum elaeagnifolium*, and *Xanthium strumarium*, do not appear on the list of dominant plants because they represented less than 0.5% of the botanical composition of the areas. Apiary 4 had a greater diversity of plants, mostly annual. Of the species present in the sites of Apiaries 4 and 5, only those with the highest floral production were quantified. While the grasses were the most abundant species, they did not present flowering during the study period of spring and early summer. This is mainly because they belong to the C4 group of plants, i.e., summer plants that flower in July or later, depending on the environmental conditions, especially precipitation [36,37]. On the other hand, most leguminous shrubs are C3 plants or winter plants that flower in spring [38].

### 3.3. Foraging and Environmental Parameters

#### 3.3.1. Shrublands

It is generally known that the foraging and other activities of honeybees are controlled or changed by weather conditions [19,39]. In this study, the analysis of the relationship between temperature and foraging showed that air temperature explained foraging in Apiaries 1 and 2 in the shrublands by 87 and 80% (R^2^), respectively (Table 3). This is in agreement with Clarke and Robert [40], who mentioned that 78% of the observed variation in bee activity was explained by temperature variations; these authors also included solar radiation as a factor affecting bee activity. Gebremedhn et al. [41] likewise reported a significant correlation coefficient between flight activity and temperature.

**Figure 2:**
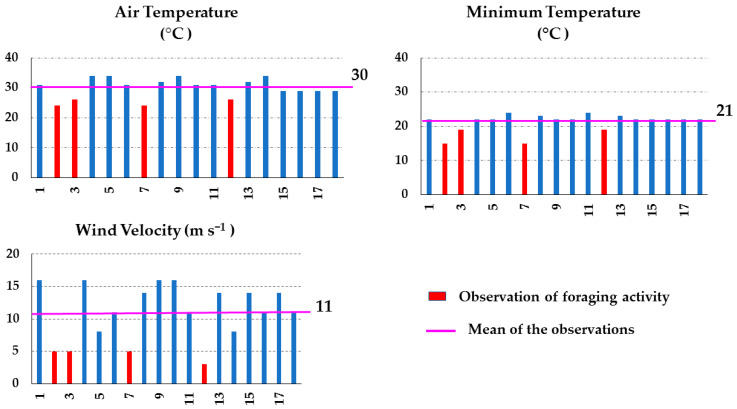
Air temperature, minimum temperature, and wind velocity data recorded during 18 samplings carried out in Apiaries 1 and 2, located in arid shrublands in Chihuahua, Northern Mexico, during spring–summer 2022.

The influence of temperature reported by previous studies is not conclusive. Vicens and Bosch [42] commented that different bee species prefer to forage at different temperatures. The foraging activity of *A. mellifera* bees has been reported as starting at temperatures averaging at 6.57 °C, according to Tan et al. [43], and at 16 °C, according to Joshi and Joshi [44]. The highest activity was recorded at temperatures of about 20 °C [43] and the lowest at 43 °C [45] as well as at or below 10 °C [44]. In the present study, foraging was observed (Figure 2) on days with temperatures between 15–17 °C above the threshold value of 9 °C [46].

Furthermore, our analysis showed that minimum temperature explained 80% of foraging (Table 3), which occurred when the minimum temperatures were between 15 and 19 °C (Figure 2). Nights with a minimum temperature of <20 °C increased foraging activity during the day. Considering the temperature projections for the study area, which estimate an increase in minimum temperature of 1.2 to 1.4 °C during 2021–2040 [47], the nights will tend to be hotter, which may affect the activity of the bees. According to various studies (e.g., [48,49]), climate change, especially changes in temperature and precipitation, threatens many animal species, including bees. The monitoring of bee colonies during the beekeeping season can aid in the study of the effects of extreme temperatures and reduced precipitation on the environment. Standardized small colonies, which are more sensitive to environmental changes, can be particularly useful in this regard [10].

Wind velocity also explained foraging, although at a lower percentage (68%) (Table 3). Foraging occurred in shrublands when the recorded wind velocity was equal to or less than 5 m s^−1^ (Figure 2). This indicates that despite the fact that wind velocity explained only 68% of foraging, this environmental parameter played an important role in bee activity. In relation to relative humidity, our results agree with those of Joshi and Joshi [44], indicating that relative humidity had less of an effect on flight activity. We observed that in shrubland areas, the relative humidity explained 41% of honeybee foraging.

The parameters of degree of cloudiness and atmospheric pressure did not show significance (Table 3) in the definition of foraging in the shrubland sites of Apiaries 1 and 2 during the sampling season.

**Figure 3:**
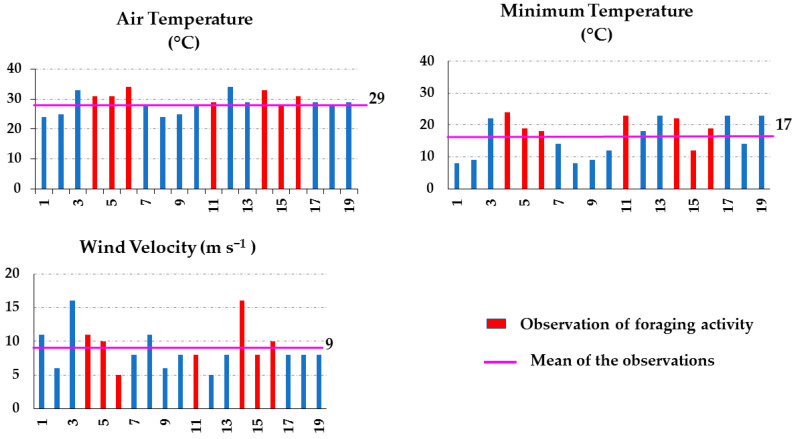
Data on air temperature, minimum temperature, and wind velocity recorded during 19 samplings carried out in Apiary 3, located in an arid shrubland area in Chihuahua, Northern Mexico, during spring–summer 2022.

The location of Apiary 3 seemed to have played an important role in honeybee activity since none of the environmental parameters significantly influenced foraging in the area. It was observed that foraging occurred even in air temperatures of up to 25 °C above the threshold value of 9 °C and with a wind velocity of 16 m s^−1^ (Figure 3). As previously mentioned, this apiary was located at a distance of 180 m from the perimeter fence of a rural community, where there could have been a favorable microenvironment for honeybee foraging. These results support the assertion of Steffan and Kuhun [50] and others that the complexity of the landscape influences the activity of foraging.

#### 3.3.2. Grasslands

**Figure 4:**
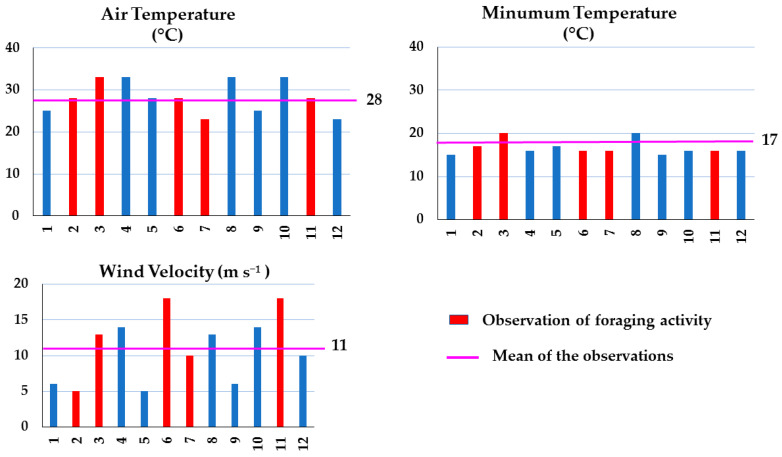
Air temperature, minimum temperature, and wind velocity data recorded during 12 samplings carried out in Apiary 4, located in arid grasslands in Chihuahua, Northern Mexico, during spring–summer 2022.

**Figure 5:**
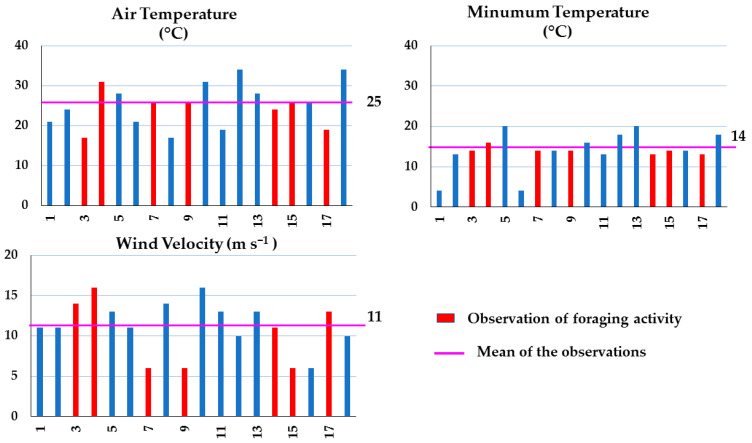
Air temperature, minimum temperature, and wind velocity data recorded during 18 samplings carried out in Apiary 5, located in arid grasslands in Chihuahua, Northern Mexico, during spring–summer 2022.

Contrary to what was observed in the shrubland sites of Apiary 1 and 2, the two apiaries located in grassland areas, i.e., Apiary 4 (Figure 4) and Apiary 5 (Figure 5), did not show a significant correlation between the environmental parameters and foraging. It was observed that foraging occurred even in air temperatures of up to 33 °C and with a wind velocity of 18 m s^−1^ (Figure 4). Again, this is possibly due to the location of the apiaries. As mentioned previously, the grassland apiaries were located at the base of a steep, narrow valley surrounded by hills that reached up to 2020 amsl. Similar to the case of Apiary 3 in the shrublands, the physiographic formation may have had a positive influence on foraging.

While the importance of the influence of meteorological elements on honeybee flight activity is well-recognized [46], our study results showed that their influence is strongly determined by the geographic location of the apiary. In our study, it was only in the apiaries established in open areas (Apiaries 1 and 2) that environmental elements such as air and minimum temperatures, wind velocity, and relative humidity showed a significant influence on honeybee activity.

### 3.4. Foraging and Apiary Distance

Previous studies have shown that the foraging distance for bee colonies in the same region may vary according to the size and strength of the colony, the type of foraging tasks, and the complexity of the landscape [9,33,50,51]. In the present study, the distance of the apiaries from the flowering areas did not show a significant influence on the intensity of foraging in the three shrubland sites. In fact, some of the lowest intensities (2 = 0.1 to 1 bees m^−2^) were recorded at the shortest distance, i.e., 250 m. On the other hand, at distances of 750 m and 1400 m, the intensity of foraging was 3 to 4 (one to three bees m^−2^). Similar results were seen in the two apiaries located in grassland areas. The distance of the apiaries from the flowering areas also did not show a significant influence on the intensity of foraging, which had a value of up to 5 (nine bees m^−2^ at distances of 750 m and four bees m^−2^ at 1400 m). The study results are in agreement with those of Otto et al. [52], who found no evidence that distance to the nearest apiary affected honeybee foraging, as they were also likely to forage far from the apiary (e.g., 7.5 km).

### 3.5. Foraging and Vegetation

#### 3.5.1. Shrublands

**Figure 6:**
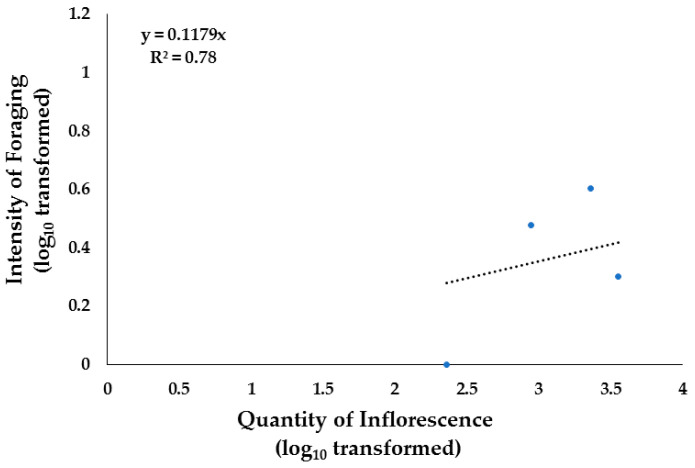
Linear regression analysis of the quantity of inflorescence (number of flowers 100 m^−2^) and intensity of foraging (Number of bees m^−2^) in Apiaries 1 and 2, located in arid shrublands areas in Chihuahua, Northern Mexico, during spring–summer 2022.

In Apiaries 1 and 2, the quantity of inflorescence significantly explained the intensity of foraging by 78% (Figure 6). *Koeberlinia spinosa and Mimosa aculeaticarpa* were preferred by honeybees. It has been previously established that the availability of suitable plant resources has an impact on foraging and that bees prefer some resources [9]. This is true of the plant species *Koeberlinia spinosa*, which represented only 1.8% of the botanical composition of the area (Table 1) but registered a high intensity of foraging (more than three bees m^−2^) in this study. On the other hand, for *Neltuma glandulosa*, which represented 48% of the botanical composition, the foraging intensity was one bee m^−2^ on average during the study period. As explained by Quinlan et al. [53], pollen forager decision-making throughout the season may be influenced by different factors, such as colony needs and forage community availability changes. These authors observed that colonies collected more pollen from plants that grew in greater abundance in flowering habitats in early July and early September, while in late July through August, a greater proportion was collected from high-protein plants, regardless of abundance.

In the case of Apiary 3, the transformed data on the quantity of inflorescence and the intensity of foraging were discarded because the Shapiro–Wilk test showed a significant departure from normality, *W*(14) = 0.79, *p* = 0.004. One possible reason for this is that, as observed by Amaya-Márquez [54], honeybees tend to exhibit high flower fidelity during foraging events and may forage on a single forb species. Mattu et al. [55] have likewise mentioned that pollen and resin bees may prefer some resources to others. In the present study, it was observed that despite the quantity of inflorescence available in the area (seven native species with floral production), the effect of plant preference was not as evident as in Apiaries 1 and 2, possibly because the intensity of foraging was distributed among some of the species present.

#### 3.5.2. Grasslands

**Figure 7:**
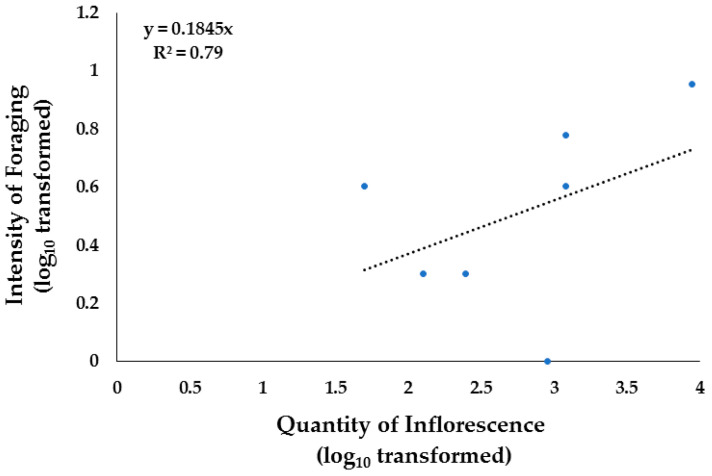
Linear regression analysis of quantity of inflorescence (number of flowers 100 m^−2^) and intensity of foraging (Number of bees m^−2^) in Apiary 4, located in arid grasslands areas in Chihuahua, Northern Mexico, during spring–summer 2022.

**Figure 8:**
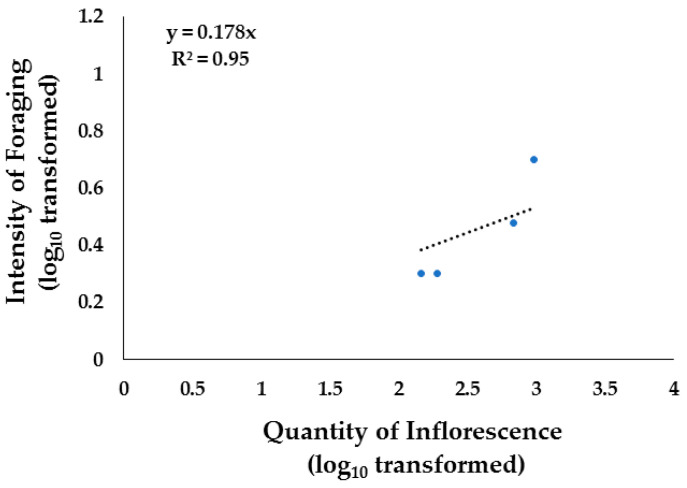
Linear regression analysis of quantity of inflorescence (number of flowers 100 m^−2^) and intensity of foraging (Number of bees m^−2^) in Apiary 5, located in arid grasslands areas in Chihuahua, Northern Mexico, during spring–summer 2022.

In the grassland sites, the species that registered a high intensity of foraging were *Mimosa aculeaticarpa* and *Neltuma glandulosa.* The quantity of inflorescence in the grassland sites explained the intensity of foraging in native vegetation by 79% in Apiary 4 (Figure 7) and by 95% in Apiary 5 (Figure 8). One possible factor influencing these results is that, as previously mentioned, Apiary 4 was close to fields where an annual bean crop was grown, while Apiary 5 depended mainly on the flowering of native vegetation present in the valley and hills.

Considering the integrated information from the grassland apiaries, the quantity of inflorescences explained the intensity of foraging in the grassland areas by 84%.

## 4. Conclusions

This study provided baseline information and findings on the influence of out-colony factors on honeybee foraging in shrublands and grasslands in the arid northern part of Mexico. In all sites, the distance of the apiary from the inflorescence sites did not show a significant influence on the intensity of foraging of honeybees. In apiaries located in open areas in shrublands, the environmental parameters air and minimum temperature explained foraging by 87% and 80% (R^2^), respectively. The other environmental parameters, wind velocity and relative humidity, explained foraging by 68 and 41%, respectively. However, these environmental factors did not significantly influence foraging in the grassland areas, where the apiaries were located in a valley surrounded by hills with altitudes ranging from 1820 to 2020 amsl. This demonstrates that the landscape and location of the colony are decisive factors in the response of honeybees to environmental factors. Our results also showed that nights with a minimum temperature of <20 °C increased the foraging activity of *A. mellifera* bees during the day. Minimum temperature is a driving variable that can be considered for further studies on the impact of climate change on bee activity.

The quantity of available inflorescence explained the intensity of foraging by 78% in shrublands and 84% in grasslands. Moreover, when honeybees depended mainly on native vegetation in grasslands, the quantity of inflorescence explained the intensity of foraging by 95%. During the study period, the preference of honeybees for certain inflorescences was evident in both shrublands and grasslands. High intensity of honeybee foraging was observed in allthorn (*Koeberlinia spinosa*) and wait-a-minute bush (*Mimosa aculeaticarpa*) in shrublands and honey mesquite (*Neltuma glandulosa*) and wait-a-minute bush (*Mimosa aculeaticarpa*) in grasslands.

To our knowledge, this study is the first of its kind conducted in an arid area in Mexico. Although it is not an area with high honey production, rural residents have shown an increasing interest in apiculture because it represents productive diversification with low starting costs. Understanding the factors influencing honeybee activity will undoubtedly help in making decisions for the best location and/or management of apiaries so as to increase apiary productivity and other agricultural and ecological benefits.

## Figures and Tables

**Table 1:** Botanical composition of species in shrubland sites with three apiaries in Chihuahua, Northern Mexico, during spring–summer 2022.

Scientific Name	Botanical Composition (%) Apiary Number		
	1	2	3
*Aloyisia gratissima*	3.5	3.6	
*Celtis pallida*	4.7		
*Condalia ericoides*			3.0
*Echinocereus stramineus*			0.6
*Flourensia cernua*	16.8	19.0	20.7
*Fouquieria splendens*			0.9
*Jatropha dioica*			4.8
*Koeberlinia spinosa*		1.8	2.7
*Larrea tridentata*	15.3	17.3	14.4
*Mimosa aculeaticarpa*	1.2	6.0	
*Neltuma glandulosa*	50.1	45.8	48.0
*Vachellia constrica*	1.5	1.8	1.2
*Vachellia biaciculata*		1.8	0.9
*Valchellia wrightii*	5.0		1.1
Others	1.9	2.9	1.7

**Table 2:** Botanical composition of species in grassland sites with two apiaries in Chihuahua, Northern Mexico, during spring–summer 2022.

Scientific Name	Botanical Composition % Apiary Number	
	4	5
*Amaranthus palmeri*	2.1	
*Aristida adscensionis*	9.3	
*Aristida divaricata*	5.9	
*Baccharis salicifolia*		2.5
*Bothriochloa barbinodis*	0.9	
*Bouteloua curtipendula*	11.0	1.3
*Bouteloua gracilis*	16.0	39.1
*Cenchrus incertus*	0.8	
*Chloris virgata*	1.5	2.5
*Crotalaria pumila*	12.8	
*Drymaria arenarioides*		2.2
*Enneapogon desvauxii*	1.9	
*Eragrostis cilianensis*	0.9	
*Eragrostis intermedia*	9.2	
*Eragrostis lehmanniana*	0.9	
*Hilaria mutica*	3.0	0.7
*Melinis repens*	2.7	
*Mimosa aculeaticarpa*	6.8	38.8
*Neltuma glandulosa*	0.7	
*Quercus emory*		3.5
*Setaria macrostachya*	1.4	
*Solanum rostratum*		1.8
*Tribulus terrestres*	2.1	3.5
Others	10.1	4.1

**Table 3:** Linear regression analysis between environmental parameters and honeybee foraging in the shrubland sites of Apiaries 1 and 2 in Chihuahua, Northern Mexico, during spring–summer 2022.

Environmental Parameter	Regression Equation	R^2^	Probability Value
Air temperature (°C)	y = −0.11x + 3.5	0.87	0.0001
Minimum temperature (°C)	y = −0.15x + 3.33	0.80	0.0001
Wind velocity (ms ^−1^)	y = −0.008x + 1.1	0.68	0.0003
Relative humidity (%)	y = −0.03x + 0.96	0.41	0.002
Cloudiness	y = 0.17x + 0.25	0.09	0.29
Atmospheric pressure (mbar)	y = −0.0006x + 0.81	0.006	0.97

## Data Availability

Data are available upon request from the corresponding author.
